# Differences in plasma lipoprotein profiles between patients with chronic peripheral neuropathic pain and healthy controls: an exploratory pilot study

**DOI:** 10.1097/PR9.0000000000001036

**Published:** 2022-09-22

**Authors:** Mika Jönsson, Emmanuel Bäckryd, Lena Jonasson, Björn Gerdle, Bijar Ghafouri

**Affiliations:** a Pain and Rehabilitation Centre, and Department of Health, Medicine and Caring Sciences, Linköping University, Linköping, Sweden; b Unit of Cardiovascular Sciences, Division of Diagnostics and Specialist Medicine, Linköping University, Linköping, Sweden

**Keywords:** Biomarker, Inflammation, VLDL, LDL, IDL, HDL, Cytokines

## Abstract

**Introduction:**

Little is still known about the underlying mechanisms that drive and maintain neuropathic pain (NeuP). Recently, lipids have been implicated as endogenous proalgesic ligands affecting onset and maintenance of pain; however, in the case of NeuP, the relationship is largely unexplored.

**Objectives:**

The aim of this study was to investigate the lipoprotein profile in patients with chronic peripheral NeuP compared with healthy controls.

**Methods:**

The concentrations of 112 lipoprotein fractions in plasma from patients with NeuP (n = 16) and healthy controls (n = 13) were analyzed using proton nuclear magnetic resonance spectroscopy. A multiplex immunoassay based on an electrochemiluminescent detection method was used to measure the concentration of 71 cytokines in plasma from patients with NeuP (n = 10) and healthy controls (n = 11). Multivariate data analysis was used to identify patterns of protein intercorrelations and proteins significant for group discrimination.

**Results:**

We found 23 lipoproteins that were significantly upregulated in patients with NeuP compared with healthy controls. When the influence of cytokines was included in a regression model, 30 proteins (8 cytokines and 22 lipoprotein fractions) were significantly upregulated or downregulated in patients with NeuP. Both conditions presented lipoprotein profiles consistent with inflammation. Body mass index did not affect lipoprotein profiles in either group. No relationship between age and lipoprotein pattern was found in NeuP, but a significant relationship was found in healthy controls.

**Conclusion:**

Patients with NeuP presented a lipoprotein profile consistent with systemic low-grade inflammation, like that seen in autoimmune, cardiometabolic, and neuroprogressive diseases. These preliminary results emphasize the importance of chronic low-grade inflammation in NeuP.

## Abstract 2

Supplemental Digital Content is Available in the Text.

Lipoprotein profiles were significantly different between patients with chronic peripheral neuropathic pain and healthy controls, indicative of ongoing systemic low-grade inflammation among the patients.

## 1. Introduction

Neuropathic pain (NeuP) is defined as pain caused by a lesion or disease in the somatosensory system, either centrally or peripherally.^5^ Traditionally, much attention in NeuP pathophysiology has been on neuronal mechanisms, such as neuronal excitability, ectopic discharge, and central and peripheral sensitization.^19,34^ However, it is now recognized that there is significant crosstalk between the nervous system and immune cells, where dysregulation in the homeostatic reciprocal communication is believed to play a pivotal role in the instigation and chronification of several chronic pain states, including NeuP.^6,16,38,46^

There is much evidence corroborating bidirectional interactions between acute-phase inflammation and pain,^22,39^ where most studies have investigated inflammatory biomolecules, such as cytokines, chemokines, and prostaglandins.^32^ Although crosstalk between inflammatory responses and neuronal cells has gained much attention recently, there is also considerable crosstalk between immune cells and lipoproteins with potential albeit less explored implications regarding nociception. This interaction between lipoproteins and the immune system can be illustrated by cytokines, which are inflammatory proteins of low molecular weight, with multifunctional and pleiotropic actions that regulate the innate and adaptive immune responses.^1,39^ Apart from cytokine-related regulatory actions in the immune system, cytokines also induce several modifications in both lipid composition and lipoprotein metabolism during inflammation.^26^ Notably, lipoproteins are mostly known for their involvement in the development of atherosclerosis and cardiovascular disease (CVD); however, oxidized lipoproteins and lipids have recently been implicated in nociception,^32,35^ thereby linking lipoproteins, lipid dysfunction, inflammatory, and nociceptive mechanisms.

Lipids are not soluble in blood and are subsequently organized into lipoproteins that are spherical macromolecular complexes of lipid and protein consisting of a highly hydrophobic core of nonpolar lipids (ie, triglycerides, cholesterol esters, and few fat-soluble vitamins) and a surface of amphipathic lipids, such as phospholipids and free cholesterol.^12^ The monolayer of lipoproteins, with polar groups oriented towards the aqueous environment and hydrophobic ends facing the core, is stabilized by enzyme cofactors called apolipoproteins (Apos).^12^ Lipoproteins are commonly classified according to their density and are divided into 5 major classes that differ in Apo content and chemical composition: chylomicrons, very-low-density lipoproteins (VLDLs), intermediate-density lipoproteins, low-density lipoproteins (LDLs), and high-density lipoproteins (HDLs).^12,28^

Lipoproteins and lipid metabolism are implicated in a variety of cellular processes ranging from a source of energy to affecting the regulation of several cell signaling processes, such as cell growth, proliferation, differentiation, apoptosis, inflammation, and membrane homeostasis. Given that bioactive lipid molecules generated from lipid metabolism can activate and regulate multiple signaling pathways, among which is nociception, dysregulation in lipid metabolism has been implicated in the development and progression of several diseases where persistent underlying inflammation is present.^21,36^ Notably, inflammation triggers some consistent metabolic alterations in lipid and lipoprotein levels, among which are increased triglyceride levels because of increased VLDL levels, increased small dense LDL, decreased LDL, and decreased HDL levels.^13,26^ Although acute inflammation-induced changes in lipid and lipoprotein metabolism occur to initially protect the organism from harmful stimuli, chronification of such unresolved inflammatory manifestations is deleterious leading to further endothelial dysfunction as a result of loss of tissue homeostasis.^26,41^ This chronification of inflammatory responses seems to be an underlying mechanism in the onset and development of several disease states including CVD, atherosclerosis, diabetes, rheumatoid arthritis, as well as in chronification of pain.^32,41^

We have previously demonstrated significantly upregulated chemokines and cytokines in patients with chronic peripheral NeuP compared with healthy controls,^4,25^ as well as a significant correlation between pain intensity and plasma cytokines and chemokines in patients with NeuP.^25^ These results suggested an activated inflammatory network in patient with chronic peripheral NeuP; however, whether this inflammatory network further extends to lipoprotein fractions is largely unexplored. The aim of this study was to investigate the lipoprotein signature in patients with chronic peripheral NeuP compared with healthy controls and to further explore the relationship between previously investigated inflammatory markers and lipoprotein fractions.

## 2. Methods

### 2.1. Patients

Patients with chronic peripheral NeuP were recruited from a clinical trial of intrathecal bolus injection of the analgesic ziconotide (ClinicalTrials.gov identifier NCT01373983). The inclusion criteria for participation were ≥ 18 years of age; chronic (ie, ≥ 6 months) peripheral NeuP follow-on surgery or trauma, where conventional pharmacologic treatment was ineffective; average pain intensity last week according to a visual analogue scale ≥ 40 mm^11^; signed informed consent; and capability of judgment, that is, the patient had to understand information pertaining to the drug, the mode of administration, and evaluate efficacy and/or side effects. After informed consent, a medical examination was performed including a basic neurological examination with a focus on different sensory modalities (light touch, pinprick, and temperature), and the following data were registered: pain diagnosis, pain duration, present and past medical history, and concomitant medication. Following the criteria published by Treede et al.,^40^ all patients had at least probable posttraumatic/postsurgical NeuP. Exclusion criteria and information about healthy controls have been published in detail elsewhere and will not be described here.^3^ An overview of patients' characteristics is presented in Table 1.

**Table 1:** Characteristics of patients with neuropathic pain; for clinical data, values outside of the reference interval are shown in bold. Liver values are indicated by p-bilirubin, p-APL, p-AST, and p-ALT and kidney values by p-CREA and p-CK for muscle tissue damage.

ICD-10	VASPI (0–100 mm)	Duration (mo)	Sensory examination	p-CREA	p-bilirubin	P-ALP	P-AST	P-ALT	P-CK	Comorbidities
S342 and G629	75	120	1, 3	87	4	1.4	0.41	0.48	3.4	History of alcohol dependency, psoriasis, tension-type headache
S342	72	39	2, 4	66	13	**0.51**	0.48	0.48	**5.9**	Polymyalgia rheumatica, hypertension
S740	82	120	1, 3	62	7	1.4	0.41	0.48	1	Ortostatism, vertebral compressions
S549	64	300	1, 2, 3	64	7	1.1	0.38	0.51	3.5	NA
S949	60	58	1, 3	86	14	1.5	0.45	0.56	1.4	NA
S342	72	72	Normal	79	12	1.2	—	0.45	**5.1**	Psoriasis, hypertension
S342	59	60	1	**133**	11	1.2	1.1	0.51	**8.4**	Mild angina, mild obstructive lung disease
S342	87	36	1, 3	47	6	0.8	0.51	0.29	3	Hypertension, anemia, dyspepsia
S342	40	120	1	70	14	1.3	0.35	0.34	1.8	NA
S343	78	79	1, 3	72	6	1	0.41	0.36	0.63	NA
S342	71	180	1	76	<3	**2**	0.33	0.33	3.5	Diabetes, mild angina, autonomic neuropathy, panic anxiety
S142	58	12	2, 4, 6	85	6	1	0.48	0.44	2.6	Fibromyalgia
S142	58	18	1	76	5	1.2	0.36	0.56	2.3	NA
S142	84	18	Normal	77	15	1.8	0.44	0.42	**7.5**	Hypertension
S342	90	84	1, 3	**100**	7	0.64	0.38	0.26	2	NA
S841	94	52	1, 4	92	10	0.71	0.58	0.3	2	NA

Reference intervals: blood tests		
	Female	Male
P-CREA	45–90	60–105
P-Bilirubin	<26	<26
P-ALP	0.6–1.8	0.6–1.8
P-AST	<0.61	<0.76
P-ALT	<0.76	<1.2
P-CK	<3.6	<6.8

Sensory examination: 1—hypoaesthesia, 2—hyperaesthesia, 3—hypoalgesia, 4—hyperalgesia, 5—allodynia, and 6—dysaesthesia.

G629, polyneuropathy, unspecified; *ICD-10*, *International Statistical Classification of Diseases*; p-ALP, alkaline phosphatase; p-ALT, alanine aminotransferase; p-AST, aspartate aminotransferase; p-CK, creatine kinase; p-CREA, creatinine; S142, injury of nerve root of cervical spine; S342, injury of nerve root of lumbar and sacral spine; S549, injury of unspecified nerve at forearm level; S740, injury of sciatic nerve at hip and thigh level; S841, injury of peroneal nerve at lower leg level; S949, injury of unspecified nerve at ankle and foot level; NA, not applicable; VASPI, visual analogue scale for pain intensity last wk.

### 2.2. Healthy controls

In short, the recruitment process of healthy controls was conducted by local advertisement at the Faculty of Medicine and Health Sciences, Linköping University, Sweden. Healthy subjects from previous studies were also contacted and asked for their participation. After informed consent, the participants underwent a structured interview to ensure the absence of any significant medical condition.

### 2.3. Ethics

The study followed the Declaration of Helsinki and Good Clinical Practice and was approved by the Ethical Review Board Regional Ethics Committee in Linköping (Dnr M136-06 and Dnr 2012/94-32). Verbal and written information was given to all participants, and written informed consent was collected from all participating subjects.

### 2.4. Sample collection

A venous blood sample of 10 mL was collected in an ethylenediaminetetraacetic acid (EDTA) tube. The samples were immediately cooled on ice and transported to Painomics laboratory, Linköping University Hospital. Each sample was then centrifuged, divided into aliquots, and stored at −76°C. The frozen samples were then transported to the nuclear magnetic resonance facility in Gothenburg for lipoprotein analysis. Cytokine analysis was performed on site in Painomics laboratory, Linköping University Hospital.

### 2.5. Lipoprotein profile

Quantitative analysis of circulating lipoproteins was performed at the nuclear magnetic resonance facility at Gothenburg using a Bruker Avance III 600 MHz spectrometer.^27,42^ Plasma samples from 16 patients and 13 healthy subjects were available for lipoprotein profile analysis. The analysis was performed according to the Bruker in vitro diagnostics research standard operating procedures (https://www.bruker.com/products/mr/nmr/avance-ivdr/overview.html). Lipoprotein fractions were obtained using Bruker in vitro diagnostics research methods.^23^ For information about principal lipoprotein fractions, refer to Supplemental Table 1, and for the list of measured lipoprotein fractions/subfractions, refer to Supplemental Table 2 (available at http://links.lww.com/PR9/A171).

### 2.6. Inflammatory profile

The analysis of inflammatory markers has been described previously.^25^ In brief, a U-PLEX assay based on an electrochemiluminescent detection method (Meso Scale Diagnostics, Rockville, MD) was used to analyze the concentrations of 71 cytokines in plasma samples from patients (n = 13) with peripheral NeuP and healthy controls (n = 13). A MESO QUICKPLEX SQ 120 instrument equipped with DISCOVERY WORKBENCH data analysis software (Meso Scale Diagnostics, Rockville, MD) was used to collect and analyze data. Electrochemiluminescent signals from calibrators were fitted to a weighted 4-parametric logistic model to form standard curves.

### 2.7. Statistics

In the omics-field, data sets often have low subject-to-variable ratios with high degrees of intercorrelations between the variables, thereby rendering traditional multivariate statistical methods (eg, multiple and logistic regression) inadequate for the purpose. Subsequently, we used advanced multivariate data analysis by projection using SIMCA-P+ and followed the recommendations presented by Wheelock and Wheelock.^45^ The data were initially scrutinized by unsupervised principal component analysis (PCA), which organizes and isolates relevant information from background noise. Potential multivariate outliers were identified by Hotelling T2 and distance to model in X-space (DModX). Loading plots show how variables are correlated. Two variables with a positive correlation will be depicted close to each other, whereas a negative correlation is depicted by the variable's dots being located on opposite sides of the origin of the plot. Orthogonal partial least squares (OPLS) discriminant analysis (OPLS-DA) was used to regress group belonging, that is, determining which lipoprotein fractions, cytokines, or chemokines were important for class differences between patients and healthy controls. As previously described, the OPLS-DA and OPLS analyses were performed in 2 steps where proteins with variable influence on projection (VIP) ≥ 1 and absolute *P*(corr) > 0.5 from the initial model were used in a second regression model.^44^ The new *R*^2^, Q^2^, and analysis of variance of cross-validated predictive residuals (CV-ANOVA), from the second model, were then presented in the results. The VIP value was used to measure the importance of each variable, where VIP ≥ 1.0 was considered significant.^11^ Comparably, an absolute *P*(corr) > 0.4 to 0.5 is generally considered significant and denotes the loading of each variable scaled as a correlation coefficient that is comparable between models. In OPLS analysis, *R*^2^ describes the goodness of fit, whereas Q^2^ describes the goodness of prediction. *R*^2^ should not be considerably greater than Q^2^; if there is a difference of >0.3 between *R*^2^ and Q^2^, it means that the robustness of the model is poor and overfitting is most likely taking place.^43,45^ To provide a familiar *P*-value metric, CV-ANOVA was used, which is a SIMCA-P+ diagnostic tool for assessing model reliability. To run the regression analysis of class-discriminating lipoproteins and inflammation-related proteins, samples from the same subjects that had undergone both methods (proton nuclear magnetic resonance and Meso Scale Discovery (MSD) assay) were included in the statistical analysis.

IBM SPSS (version 24.0; IBM Corporation, Route 100 Somers, NY) and SIMCA-P+ (version 17.0; Sartorius Stedim Biotech, Umeå, Sweden) were used for all statistical analyses, and *P* ≤ 0.05 was set as a level of significance. IBM SPSS was used for all descriptive statistics, and results were given as mean values. For comparisons between groups, the Mann–Whitney *U* test was conducted.

## 3. Results

### 3.1. Demographic data over patients with neuropathic pain and healthy controls

Basic demographic data about patients and healthy controls are summarized in Table 2. There was a significant difference between the 2 groups for age and body mass index (BMI), but there was no significant difference in sex distribution between the 2 groups (Table 2).

**Table 2:** Basic demographic data over patients and healthy controls.

Variables	Patients (n = 16)	Healthy controls (n = 13)	Statistics *P*
Age (y)	57 (39–75)	43 (21–57)	0.01
Sex (%female)	38	46	0.39
BMI (kg/m^2^)	26.90 (20.20–32.41)	23.60 (19.50–27.60)	0.02

The data are shown as median (range), with statistical comparisons between patients and healthy controls shown furthest to the right.

BMI, body mass index.

### 3.2. Regression of class-discriminating lipoproteins

**Figure 1.**
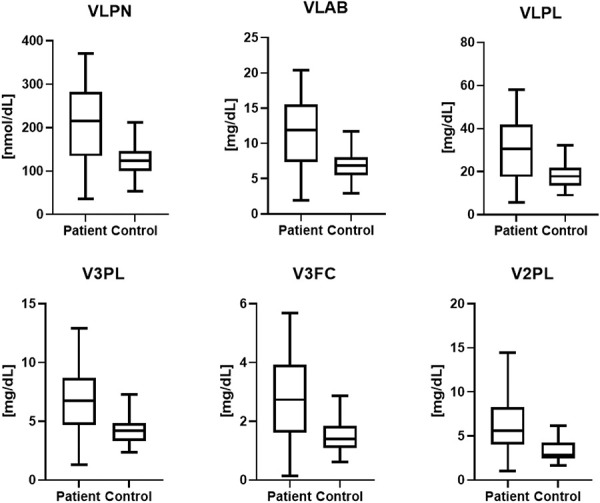
Lipoprotein concentrations in patients and healthy controls for the 6 most significant fractions according to OPLS-DA. Median values are represented by horizontal lines, and boxes represent the interquartile range. Minimum and maximum values are represented by the ends of the whiskers. VLPN (*P* = 0.01), VLAB (*P* = 0.01), VLPL (*P* = 0.02), V3PL (*P* = 0.02), V3FC (*P* = 0.02), and V2PL (*P* = 0.02). OPLS-DA, orthogonal partial least squares discriminant analysis

A total of 112 lipoproteins were analyzed. An unsupervised PCA was performed, and no strong outliers or serious moderate outliers were identified (n = 29, 4 principal components (PCs), *R*^2^ = 0.86, Q^2^ = 0.71). A significant OPLS-DA regression model was obtained which discriminated lipoproteins between patients with NeuP (n = 16) and healthy controls (n = 13), 112 *X*-variables, *R*^2^ = 0.28, Q^2^ = 0.21, and *P* = 0.05 by CV-ANOVA. A combination of absolute *P*(corr) > 0.5 and VIP > 1 was used as cutoff points, giving 23 of 112 lipoprotein fractions identified as significant for group separation (Table 3). From Table 3, 6 lipoprotein fractions with the highest absolute *P*(corr) were selected (VLPN, VLAB, VLPL, V3PL, V3FC, and V2PL). The concentration of each fraction was compared between patients and healthy controls and differences were illustrated as box plots (Fig. 1). A clinical case study of pain parameters with respect to lipoprotein fractions was also conducted, refer to Supplemental Figure 1, available at http://links.lww.com/PR9/A171.

**Table 3:** List of significant lipoprotein fractions and subfractions identified by orthogonal partial least squares discriminant analysis, all of which were upregulated in patients with neuropathic pain.

Lipoprotein	Description	VIP	*P*(corr)
VLPN	Particle number	1.21	−0.98
VLAB	Apo-B	1.21	−0.98
VLPL	Phospholipids	1.21	−0.98
V3PL	Phospholipids	1.20	−0.97
V3FC	Free cholesterol	1.19	−0.97
V2FC	Free cholesterol	1.19	−0.96
V2PL	Phospholipids	1.18	−0.96
VLCH	Cholesterol	1.18	−0.95
VLFC	Free cholesterol	1.17	−0.95
VLTG	Triglycerides	1.16	−0.94
V1PL	Phospholipids	1.16	−0.94
V3TG	Triglycerides	1.16	−0.94
V1CH	Cholesterol	1.16	−0.94
V2TG	Triglycerides	1.15	−0.93
V2CH	Cholesterol	1.15	−0.93
V3CH	Cholesterol	1.15	−0.93
TPTG	Total particle triglycerides	1.15	−0.93
V1TG	Triglycerides	1.14	−0.93
V1FC	Free cholesterol	1.12	−0.91
H4TG	Triglycerides	1.11	−0.90
V4TG	Triglycerides	1.10	−0.89
IDTG	IDL-triglycerides	1.09	−0.88
V4PL	Phospholipids	1.08	−0.87
R^2^	0.28		
Q^2^	0.21		
CV-ANOVA	0.05		

First letter denotes principal lipoprotein fraction: V, very-low-density lipoprotein; L, low-density lipoprotein; ID, intermediate-density lipoprotein; H, high-density lipoprotein.

Subfractions of principal lipoprotein fractions are shown by a number indicating density of that subfraction; the higher the number, the greater the density.

CV-ANOVA, analysis of variance of cross-validated predictive residuals; IDL, intermediate-density lipoproteins; VIP, variable influence of projection.

### 3.3. Regression of class-discriminating lipoproteins and inflammation-related proteins

**Figure 2.**
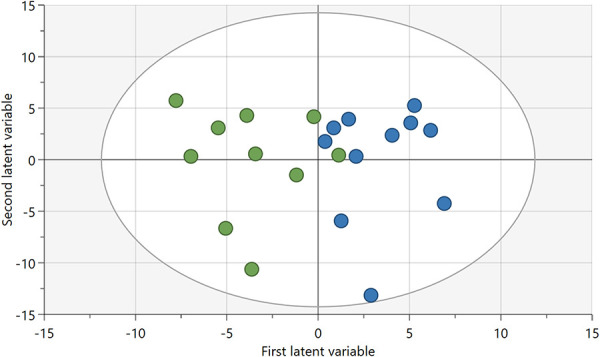
Score plot of OPLS-DA regression model of lipoproteins and cytokines among patients with NeuP (green) and healthy controls (blue). NeuP, neuropathic pain; OPLS-DA, orthogonal partial least squares discriminant analysis.

An OPLS-DA regression model was computed for patients with NeuP (n = 10) and healthy controls (n = 11), 183 *X*-variables (112 lipoprotein fractions and 71 cytokines), *R*^2^ = 0.67, Q^2^ = 0.42, and *P* = 0.06 by CV-ANOVA. The number of patients and controls was determined by participation in a previous study on inflammatory substances and thus on the availability of both lipoprotein and cytokine samples in plasma.^25^ The model is presented by a score plot (Fig. 2), and the contributing proteins are listed in Table 4. A total of 30 proteins had an absolute *P*(corr) > 0.5 and VIP > 1, of which 8 were cytokines, 20 LDL fractions, and 2 HDL fractions. Of the 30 proteins, only 5 were upregulated in patients, all of which were cytokines (MIP3β, IFNα2a, IL-18, IL1RA, and MDC).

**Table 4:** List of lipoprotein fractions/subfractions and inflammatory cytokines and chemokines contributing to the orthogonal partial least squares discriminant analysis model depicted in Figure 3.

Lipoprotein/cytokine	Description	VIP	*P*(corr)	Upregulated or downregulated in patients
MIP3β	Cytokine	1.44	−0.80	↑
IFNα2a	Cytokine	1.36	−0.75	↑
L3PL	Phospholipids	1.35	0.71	↓
L2PL	Phospholipids	1.35	0.65	↓
L2CH	Cholesterol	1.34	0.65	↓
L3CH	Cholesterol	1.34	0.70	↓
L2AB	Apo-B	1.33	0.65	↓
L2PN	Particle number	1.33	0.65	↓
L2FC	Free cholesterol	1.26	0.56	↓
IL-18	Cytokine	1.23	−0.67	↑
L3AB	Apo-B	1.22	0.64	↓
L3PN	Particle number	1.22	0.64	↓
L4CH	Cholesterol	1.22	0.66	↓
L4FC	Free cholesterol	1.17	0.64	↓
H4PL	Phospholipids	1.17	0.61	↓
GRO-α	Cytokine	1.15	0.62	↓
L1CH	Cholesterol	1.14	0.60	↓
L3FC	Free cholesterol	1.14	0.50	↓
L4PL	Phospholipids	1.14	0.62	↓
ENA-78	Cytokine	1.11	0.61	↓
L1FC	Free cholesterol	1.10	0.54	↓
L1PL	Phospholipids	1.07	0.56	↓
LDFC	Free cholesterol	1.07	0.54	↓
L4PN	Particle number	1.04	0.56	↓
L4AB	Apo-B	1.04	0.56	↓
H4A1	Apo-A1	1.04	0.53	↓
ITAC	Cytokine	1.03	0.56	↓
IL1RA	Cytokine	1.03	−0.56	↑
MDC	Cytokine	1.03	−0.57	↑
LDCH	Cholesterol	1.02	0.54	↓
R^2^	0.67			
Q^2^	0.42			
CV-ANOVA	0.06			

First letter denotes principal lipoprotein fraction: V, very-low-density lipoprotein; L, low-density lipoprotein; ID, intermediate-density lipoprotein; H, high-density lipoprotein.

Subfractions of principal lipoprotein fractions are shown by a number indicating density of that subfraction; the higher the number, the greater the density.

CV-ANOVA, analysis of variance of cross-validated predictive residuals; VIP, variable influence of projection.

### 3.4. Multivariate intercorrelations between lipoproteins and inflammatory substances

Two unsupervised PCA models, 1 for healthy controls and 1 for patients with NeuP, were computed to investigate alterations in the correlation structure of lipoproteins and inflammatory substances together in patients compared with controls.

**Figure 3.**
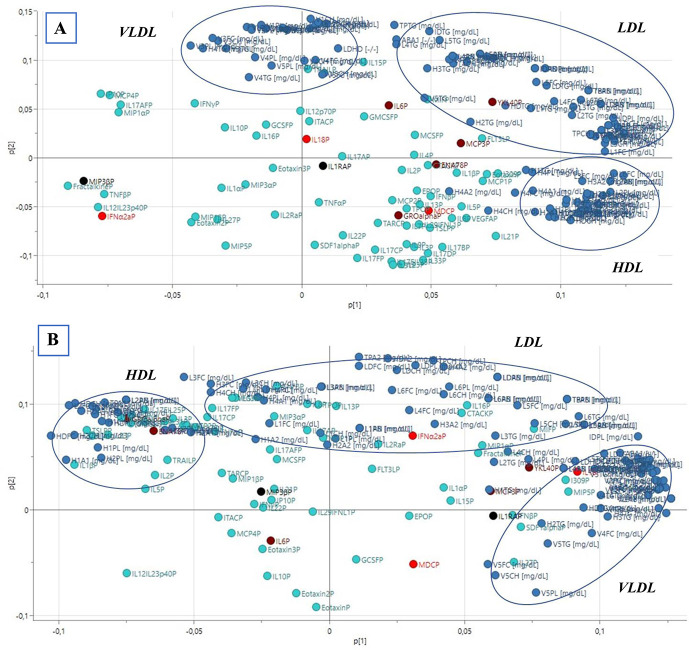
Loading plots of PCA models showing distribution of lipoproteins and cytokines in plasma from (A) healthy controls and (B) patients with NeuP. Variables with a positive correlation are depicted close to each other, whereas a negative correlation is depicted by variables being located diagonally on opposite sides of the origin of the plot. Lipoproteins are depicted by blue dots and cytokines by turquoise dots; clusters of VLDL, LDL, and HDL are shown by blue circles. Significant cytokines are depicted by red dots (IL-18, MDC, and IFNα2A), significant cytokines previously identified by our laboratory are depicted by dark red dots (IL-6, YKL-40, MCP-3, ENA-78, and GRO-α), and black dots indicate significant cytokines from current and previous result (IL1RA and MIP3β). HDL, high-density lipoproteins; LDL, low-density lipoproteins; NeuP, neuropathic pain; PCA, principal component analysis; VLDL, very-low-density lipoprotein.

**Figure 4.**
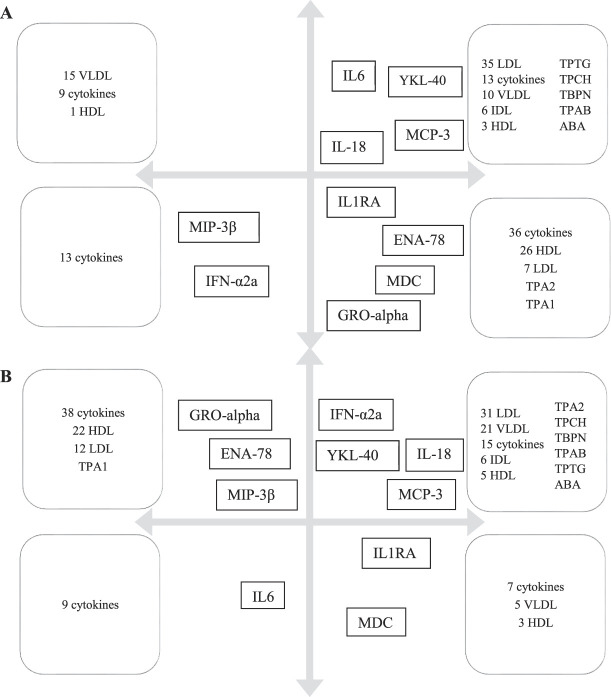
Simplified illustration of the significant cytokines depicted in the original loading plots of the 2 PCA models (Fig. 3A, B). Arrows in (A) and (B) represent the *x*-axis and *y*-axis as seen in Figures 3A and B. The significant cytokines are placed in each quadrant as they approximately appeared in the original loading plots of the 2 PCA models (Fig. 3A, B). Variables with a positive correlation are depicted close to each other, whereas a negative correlation is depicted by variables being located diagonally on opposite sides of the origin of the plot. The cytokines MIP3β, IFNα2a, IL-18, MCP, IL-6, IL1RA, MCP-3, and YKL-40 were previously shown to be upregulated in plasma from patients with NeuP, whereas ENA-78 and GRO-α were downregulated. Boxes describe the composition of lipoprotein fractions and cytokines in each quadrant of Figures 3A and B, respectively. (A) Illustration of the distribution of cytokines and lipoproteins for healthy controls seen in Figure 3A. (B) Illustration of the distribution of cytokines and lipoproteins for patients with NeuP seen in Figure 3B. HDL, high-density lipoproteins; IDL, intermediate-density lipoproteins; LDL, low-density lipoproteins; NeuP, neuropathic pain; PCA, principal component analysis; VLDL, very-low-density lipoprotein

First, an unsupervised PCA model was computed for healthy controls (n = 11); the model had 2 PCs (*R*^2^ = 0.59, Q^2^ = 0.28). No strong or serious moderate multivariate outliers were identified according to Hotelling T2 and DModX. The loading plot is shown in Figure 3A, and a schematic simplification is shown in Figure 4A. From the model, 3 distinct clusters of lipoprotein fractions could be seen for VLDL, LDL, and HDL. There was a negative correlation between most VLDL fractions (n = 15) and most HDL fractions (n = 26) (Fig. 3A and simplified in Fig. 4A). For inflammatory substances, there was a positive correlation between IL-6, YKL-40, MCP-3, IL-18, and most LDL fractions (n = 35), which were all negatively correlated to MIP3β and INFα2a. A positive correlation was also seen between IL1RA, ENA-78, GRO-α, MDC, and 26 HDL fractions, all of which were negatively correlated to 15 VLDL fractions (Fig. 3A and simplified in Fig. 4A).

Next, an unsupervised PCA model was computed for patients with NeuP (n = 10), as depicted in Figure 3B and simplified in Figure 4B. The model had 2 PCs and no strong or serious moderate multivariate outliers according to Hotelling T2 and DModX (*R*^2^ = 0.53, Q^2^ = 0.14). In similarity to the model for healthy controls, 3 distinct clusters of lipoprotein fractions could be distinguished. However, unlike healthy controls, only 5 VLDL fractions (compared with 15) showed a negative correlation to most of the HDL fractions (n = 22). Conversely, most of the VLDL fractions (n = 21) showed a positive correlation to the LDL cluster (n = 31) which was not seen among healthy controls (Fig. 3B and simplified in Fig. 4B). Moreover, the association pattern between lipoprotein fractions and inflammatory substances in plasma differed between patients and healthy controls in several aspects. For instance, unlike healthy controls, IL-6 in patients with NeuP was negatively correlated to YKL-40, IL-18, MCP-3, LDL (n = 31), and VLDL (n = 21). However, IL-6 showed a similar negative correlation to IFNα2a in both groups. Moreover, in contrast to healthy controls, GRO-α and ENA-78 were positively correlated with MIP3β among patients and were negatively correlated to IL1RA and MDC (which were positively correlated in healthy controls) (Fig. 3B and simplified in Fig. 4B). On the other hand, both ENA-78 and GRO-α were positively correlated to most HDL fractions (n = 22) in both patients and healthy controls. To summarize, although there were some similarities between patients and controls, the overall correlation pattern of lipoproteins and cytokines was disturbed in several ways in patients compared with healthy controls.

### 3.5. Effects of age and body mass index on lipoprotein profiles

Because there were significant differences between patients and controls for age and BMI (Table 2), OPLS regression models were computed to explore the influence of age and BMI, respectively, on lipoprotein profiles.

It was not possible to significantly regress BMI by using lipoproteins as predictor variables, neither in patients nor in healthy controls (CV-ANOVA = 1 and CV-ANOVA = 0.13, respectively). For age, there was no significant influence on lipoproteins by age for patients with NeuP, *R*^2^ = 0.29 Q^2^ = −0.04 and CV-ANOVA = 1. However, for healthy controls, there was a significant relationship between age and lipoproteins, *R*^2^ = 0.66 Q^2^ = 0.59 and CV-ANOVA = 0.01, see Supplemental Figure 2 for healthy controls and Supplemental Figure 3 for patients with NeuP, available at http://links.lww.com/PR9/A171.

## 4. Discussion

In this study, we have investigated the lipoprotein signature in patients with chronic peripheral NeuP compared with healthy controls. Of 112 lipoprotein fractions, 23 proteins were significantly upregulated in patients compared with healthy controls, where most consisted of fractions of VLDL. By contrast, when the influence of inflammatory substances was included in the OPLS-DA analysis, 30 proteins were found to be significantly upregulated or downregulated in patients with NeuP, where most consisted of LDL fractions. Moreover, regression analysis showed that BMI did not affect the lipoprotein profiles in either group. Similarly, no relationship between age and lipoprotein signature was found among patients; however, a significant relationship was found in healthy controls.

Inflammation and infections cause several general alterations in lipid metabolism and lipoprotein function, including increased triglycerides because of increased hepatic VLDL production/secretion, cytokine-mediated triglyceride increase and decreased clearance of triglyceride-rich lipoproteins, decreased HDL cholesterol, decreased LDL, and increased small dense LDL.^14,18^ Lipoprotein function is also adversely affected by inflammation, where reverse cholesterol transport is negatively affected, antioxidant ability of HDL is reduced, and LDL is more readily oxidized.^14^ Moreover, these abnormalities in lipoproteins and lipid metabolism are related to the severity of the underlying inflammatory disease; the more severe the underlying disease, the more consistently lipid abnormalities are observed.^14^ In corroboration with our previous study where we showed that patients with NeuP had altered inflammatory profiles in plasma and saliva, indicating low-grade systemic inflammation, the OPLS-DA model of lipoprotein fractions signified lipoprotein patterns associated with inflammation (ie, high VLDL levels and subsequently high triglyceride levels) (Table 3). However, when the influence of inflammatory substances was included in the OPLS-DA model, the resultant lipoprotein pattern changed from significantly high VLDLs in patients to significantly low LDLs (cholesterol, free cholesterol, and phospholipids) compared with healthy controls (Table 4). In addition, there was a significant decrease in apoA-1-containing HDL among patients with NeuP (Table 4). Under normal conditions, HDL has protective, anti-inflammatory functions, but during systemic inflammation or acute infection, HDL function is impaired because of specific changes to the particle which renders it proinflammatory.^2^ ApoA-1 is one such key target because it is crucial for HDL's antioxidant and anti-inflammatory properties as well as in reverse cholesterol transport.^2^ Reduced HDL and apoA-1 levels are typical during inflammation and infection,^26^ and chronic systemic inflammation is known to reduce apoA-1 levels through several mechanisms.^29^ Hence, the HDL profile in NeuP, with lower levels of apoA-1, seems supportive of a proinflammatory HDL state.

Notably, much focus on lipoprotein profiles has been in the context of CVD, where an increased risk for CVD has been associated with low levels of HDL cholesterol and high levels of total cholesterol, LDL cholesterol, and triglycerides.^9^ However, by contrast, patients with chronic systemic inflammation, such as autoinflammatory, autoimmune, and neuroprogressive diseases, show a lipid paradox that is associated with an increased risk of developing CVD.^29^ In these patients, a CVD risk is conversely associated with low total cholesterol and LDL cholesterol but in similarity with high triglyceride levels.^29^ Accordingly, the lipoprotein profile of patients with NeuP seems to be more like that of patients with chronic systemic inflammation than the traditional lipoprotein risk profile for CVD, thus emphasizing the importance of underlying low-grade inflammation in NeuP.

Notably, NeuP is rarely presented on its own but rather depicts a complex biopsychosocial entity, intertwined with several comorbidities such as anxiety, depression, insomnia, and obesity.^47^ Chronic pain and obesity are 2 such common comorbidities, with a likely mutual detrimental influence on one another, that together poses a vicious cycle and a serious concern for public health and society.^7,20,30,33^ Interestingly, in our study, there was no significant relationship between lipoproteins and BMI for neither patients nor healthy controls (CV-ANOVA = 1 and CV-ANOVA = 0.13 respectively). Although, pain and obesity are significantly associated clinical syndromes, the relationship between pain sensitivity and obesity is less clear.^33^ For instance, a cohort study of patients with chronic pain and obesity showed that pain intensity was not associated with weight change after an interdisciplinary multimodal pain rehabilitation intervention,^10^ whereas a study on patients with fibromyalgia showed that overweight patients had higher pain sensitivity compared with patients with normal weight.^31^ Given that BMI did not have a significant impact on the lipoprotein signature among patients nor healthy controls, suggests that weight per say is not the determining factor on lipoprotein composition in this study. However, further studies including subjects with normal BMI and high BMI are warranted to be able to conclude if BMI influences the lipoprotein fractions investigated in this study.

Moreover, the effect of aging on systemic alterations in lipid metabolism has been extensively studied.^8,15,24,37^ General changes in lipid metabolism during aging include changed triglyceride and lipoprotein metabolism (increased levels of plasma triglycerides and lipoproteins, decreased activity of lipoprotein lipase, and decreased postprandial plasma triglyceride clearance rates), decreased adipose tissue lipolysis, elevated ectopic fat deposition, and altered lipid transport proteins.^8,37^ However, our results showed that there was no significant relationship between age and lipoprotein fractions among patients with chronic peripheral NeuP (CV-ANOVA = 1), but a significant relationship was seen among healthy controls (CV-ANOVA = 0.01). Given that the aging process is associated with a state of chronic inflammation, known as inflammaging,^17^ it is possible that the relationship between aging and lipoprotein fractions in patients with NeuP was masked by a NeuP-related systemic low-grade inflammation that disrupted the normal relationship between age and inflammation. Nevertheless, it is important to stress that the age span for patients with NeuP in this study ranged from 39 to 75 years compared with 21 to 57 years for healthy controls, thus missing representation of a younger population. Accordingly, a greater sample size including patients belonging to the younger population would be needed to explore whether NeuP uncouples natural lipoprotein alterations from age-related changes.

### 4.1. Limitations

This study had several limitations, including low sample sizes that depended on the fact that this was an additional study conducted from samples collected before a ziconotide trial and the number of patients was calculated with respect to that trial. The ziconotide trial further required cerebrospinal fluid sampling which is an invasive procedure that limited the inclusion of both sex-matched and age-matched healthy controls, which presented a major constraint to this pilot study. The difference in sample numbers between the 2 analyses (regression of class-discriminating lipoproteins, n = 29, and regression analysis of class-discriminating lipoproteins and inflammation-related proteins, n = 21) depended on the fact that the same samples were used twice. To run the regression analysis of class-discriminating lipoproteins and inflammation-related proteins, only samples that had undergone both methods (proton nuclear magnetic resonance and MSD) were included. Unfortunately, some samples ran out and the 21 samples that had results from both methods were used. Another issue concerns the influence of patient comorbidities on lipoprotein patterns. It is possible that the lipoprotein profile presented in this study indicates a state of chronic disease rather than a state of chronic NeuP; however, given that NeuP rarely is presented in isolation, it is reflective of the patient population.

## 5. Conclusion

The importance of inflammation in the instigation and maintenance of NeuP is well recognized; however, much focus has been on inflammatory proteins, such as cytokines and chemokines. In this study, we showed that patients with chronic peripheral NeuP also presented altered lipoprotein profiles consistent with low-grade inflammation, like that seen in autoinflammatory, autoimmune, and neuroprogressive diseases. These, preliminary results present a potential for further larger metabolic studies on NeuP.

## Disclosures

The authors report no conflicts of interest.

## Appendix A. Supplemental digital content

Supplemental digital content associated with this article can be found online at http://links.lww.com/PR9/A171.

## Supplementary Material

SUPPLEMENTARY MATERIAL
