# Supplementary material for: Differences in plasma lipoprotein profiles between patients with chronic peripheral neuropathic pain and healthy controls: an exploratory pilot study
Source: Pain Rep. 2022 Sep 22;7(5):e1036. doi: 10.1097/PR9.0000000000001036 (PMC9529241; doi:10.1097/PR9.0000000000001036)
Supplement: SUPPLEMENTARY MATERIAL [file painreports-7-e1036-s001.pdf]

Supplement:

Clinical pain parameters in respect to lipoprotein fractions – clinical case study

From **Table 1**, three groups for sensory examination findings were selected: one depicting hypersensitivity and two illustrating hyposensitivity. Group A represented hypersensitivity and consisted of 2 patients that reported hyperaesthesia, hyperalgesia, and dysaesthesia. Group B and C represented cases of hyposensitivity; group B consisted of 4 patients reporting hypoaesthesia and group C consisted of 6 patients reporting hypoaesthesia and hypoalgesia. The concentration of each significant lipoprotein fraction identified by OPLS-DA, **Table 3,** was compared between the groups and presented as scatter plots. Nine graphs were selected, showing highest difference between the groups of hyposensitivity and hypersensitivity (**Supplemental Figure 1**). Generally, group A showed higher concentration of the 9 lipoprotein fractions compared to group B and C, however much larger groups are required to validate this seeming trend.

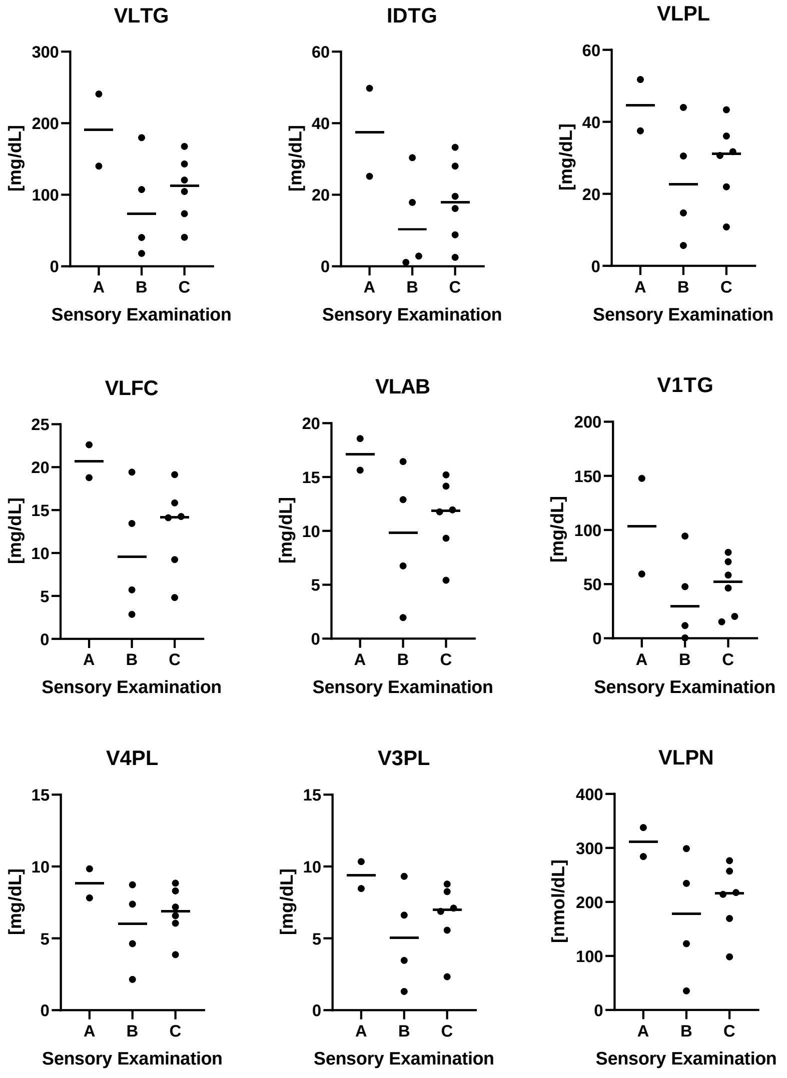

Supplemental Figure 1: *Clinical case-study: pain parameters in respect to lipoprotein fractions. Scatter plot of lipoprotein fractions associated with sensory examination. Each dot represents a patient and median values are shown by a horizontal line. Sensory examination groups:*
A – hyperaesthesia, hyperalgesia, and dysaesthesia.
B – hypoaesthesia.
C – hypoaesthesia, and hypoalgesia.

Effects of age on lipoprotein profiles

The observed vs predicted relationship between lipoprotein fractions and age is shown in **Supplemental Figure 2** for healthy controls and in **Supplemental Figure 3** for NeuP patients.

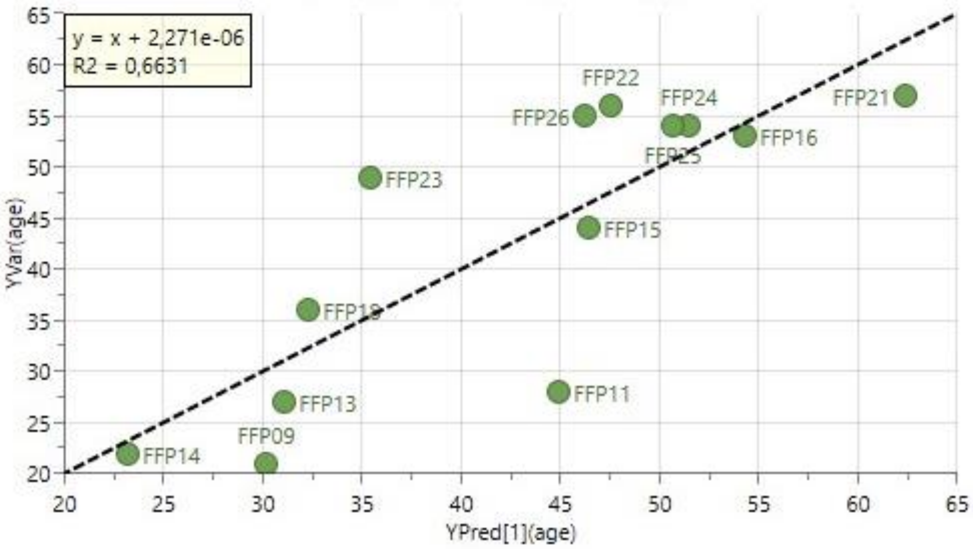

***Supplemental Figure 2**: Effects of age on lipoprotein profiles among healthy controls; observed vs predicted relationship to age, CV-ANOVA=0.018*

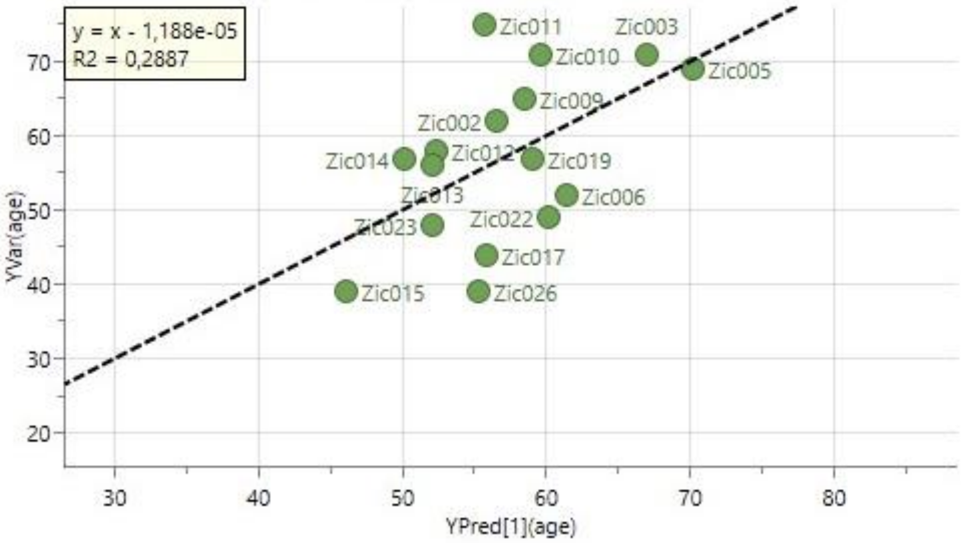

***Supplemental Figure 3**: Effects of age on lipoprotein profiles among patients with chronic peripheral NeuP; observed vs predicted relationship to age, CV-ANOVA=1.*

Supplement Table 1: Principal Lipoprotein fractions

Abbreviation	*Description*	*Density (kg/L)*
VLDL	Very low-density lipoprotein	0.950 - 1.006
LDL	Low density lipoprotein	1.006 - 1.019
IDL	Intermediate density lipoprotein	1.019 - 1.063
HDL	High density lipoprotein	1.063 - 1.210

<u>Subfractions of Principal Lipoprotein fractions (numbering indicating increasing density):</u>

VLDL – 5 subfractions (VLDL-1, VLDL-2, VLDL-3, VLDL-4, VLDL-5)

LDL – 6 subfractions (LDL-1, LDL-2, LDL-3, LDL-4, LDL-5, LDL-6)

HDL – 4 subfractions (HDL-1, HDL-2, HDL-3, HDL-4)

<u>Abbreviation of Apolipoproteins:</u>

Apo-B = Apolipoprotein B100

Apo-A = Apolipoprotein A

Supplement Table 2: All lipoprotein fractions and subfractions measured

Abbreviation	*Description*	*Subfraction*
TPTG	Total particle triglycerides	
TPCH	Total particle cholesterol	
LDCH	LDL-cholesterol	
HDCH	HDL-cholesterol	
TPA1	Total particle Apo-A1	
TPA2	Total particle Apo-A2	
TPAB	Total particle Apo-B	
LDHD	LDL-cholesterol / HDL-cholesterol	
ABA1	Apo-B / Apo-A1	
TBPN	Total Concentration of ApoB carrying Particles	
VLPN	VLDL- particle number	
IDPN	IDL-particle number	
LDPN	LDL-particle number	
L1PN	LDL1-particle number	1
L2PN	LDL2-particle number	2
L3PN	LDL3-particle number	3
L4PN	LDL4-particle number	4
L5PN	LDL5-particle number	5
L6PN	LDL6-particle number	6
VLTG	VLDL-triglycerides	
IDTG	IDL-triglycerides	

LDTG	LDL-triglycerides	
HDTG	HDL-triglycerides	
VLCH	VLDL-Cholesterol	
IDCH	IDL-Cholesterol	
VLFC	VLDL-Free Cholesterol	
IDFC	IDL-Free Cholesterol	
LDFC	LDL-Free Cholesterol	
HDFC	HDL-Free Cholesterol	
VLPL	VLDL-Phospholipids	
IDPL	IDL-Phospholipids	
LDPL	LDL-Phospholipids	
HDPL	HDL-Phospholipids	
HDA1	HDL-Apo-A1	
HDA2	HDL-Apo-A2	
VLAB	VLDL-Apo-B	
IDAB	IDL-Apo-B	
LDAB	LDL-Apo-B	
V1TG	VLDL1-Triglycerides	1
V2TG	VLDL2-Triglycerides	2
V3TG	VLDL3-Triglycerides	3
V4TG	VLDL4-Triglycerides	4
V5TG	VLDL5-Triglycerides	5
V1CH	VLDL1-Cholesterol	1
V2CH	VLDL2-Cholesterol	2
V3CH	VLDL2-Cholesterol	3
V4CH	VLDL2-Cholesterol	4
V5CH	VLDL2-Cholesterol	5
V1FC	VLDL1- Free Cholesterol	1
V2FC	VLDL2- Free Cholesterol	2
V3FC	VLDL3- Free Cholesterol	3
V4FC	VLDL4- Free Cholesterol	4
V5FC	VLDL5- Free Cholesterol	5
V1PL	VLDL1-Phospholipids	1
V2PL	VLDL2-Phospholipids	2
V3PL	VLDL3-Phospholipids	3
V4PL	VLDL4-Phospholipids	4
V5PL	VLDL5-Phospholipids	5
L1TG	LDL1-Triglycerides	1
L2TG	LDL2-Triglycerides	2
L3TG	LDL3-Triglycerides	3
L4TG	LDL4-Triglycerides	4
L5TG	LDL5-Triglycerides	5
L6TG	LDL6-Triglycerides	6
L1CH	LDL1-Cholesterol	1
L2CH	LDL2-Cholesterol	2
L3CH	LDL3-Cholesterol	3

L4CH	LDL4-Cholesterol	4
L5CH	LDL5-Cholesterol	5
L6CH	LDL6-Cholesterol	6
L1FC	LDL1-Free Cholesterol	1
L2FC	LDL2-Free Cholesterol	2
L3FC	LDL3-Free Cholesterol	3
L4FC	LDL4-Free Cholesterol	4
L5FC	LDL5-Free Cholesterol	5
L6FC	LDL6-Free Cholesterol	6
L1PL	LDL1-Phospholipids	1
L2PL	LDL2-Phospholipids	2
L3PL	LDL3-Phospholipids	3
L4PL	LDL4-Phospholipids	4
L5PL	LDL5-Phospholipids	5
L6PL	LDL6-Phospholipids	6
L1AB	LDL1-ApoB	1
L2AB	LDL2-ApoB	2
L3AB	LDL3-ApoB	3
L4AB	LDL4-ApoB	4
L5AB	LDL5-ApoB	5
L6AB	LDL6-ApoB	6
H1TG	HDL1-Triglycerides	1
H2TG	HDL2-Triglycerides	2
H3TG	HDL3-Triglycerides	3
H4TG	HDL4-Triglycerides	4
H1CH	HDL1-Cholesterol	1
H2CH	HDL2-Cholesterol	2
H3CH	HDL3-Cholesterol	3
H4CH	HDL4-Cholesterol	4
H1FC	HDL1-Free Cholesterol	1
H2FC	HDL2-Free Cholesterol	2
H3FC	HDL3-Free Cholesterol	3
H4FC	HDL4-Free Cholesterol	4
H1PL	HDL1-Phospholipids	1
H2PL	HDL2-Phospholipids	2
H3PL	HDL3-Phospholipids	3
H4PL	HDL4-Phospholipids	4
H1A1	HDL1-Apo-A1	1
H2A1	HDL2-Apo-A1	2
H3A1	HDL3-Apo-A1	3
H4A1	HDL4-Apo-A1	4
H1A2	HDL1-Apo-A2	1
H2A2	HDL2-Apo-A2	2
H3A2	HDL3-Apo-A2	3
H4A2	HDL4-Apo-A2	4
